# The Dutch national paediatric heart transplantation programme: outcomes during a 23-year period

**DOI:** 10.1007/s12471-022-01703-w

**Published:** 2022-07-15

**Authors:** Stefan Roest, Marijke H. van der Meulen, Lennie M. van Osch-Gevers, Ulrike S. Kraemer, Alina A. Constantinescu, Matthijs de Hoog, Ad J. J. C. Bogers, Olivier C. Manintveld, Pieter C. van de Woestijne, Michiel Dalinghaus

**Affiliations:** 1 Department of Paediatric Cardiology, Erasmus MC—Sophia Children’s Hospital, University Medical Centre Rotterdam, Rotterdam, The Netherlands; 2 Department of Cardiology, Thorax Centre, Erasmus MC, University Medical Centre Rotterdam, Rotterdam, The Netherlands; 3 Erasmus MC Transplant Institute, Erasmus MC, University Medical Centre Rotterdam, Rotterdam, The Netherlands; 4 Department of Paediatric Intensive Care, Erasmus MC—Sophia Children’s Hospital, University Medical Centre Rotterdam, Rotterdam, The Netherlands; 5 Department of Cardiothoracic Surgery, Erasmus MC, University Medical Centre Rotterdam, Rotterdam, The Netherlands

**Keywords:** Heart transplantation, Mortality, Children, Waiting list

## Abstract

**Background:**

Since 1998, there has been a national programme for paediatric heart transplantations (HT) in the Netherlands. In this study, we investigated waiting list mortality, survival post-HT, the incidence of common complications, and the patients’ functional status during follow-up.

**Methods:**

All children listed for HT from 1998 until October 2020 were included. Follow-up lasted until 1 January 2021. Data were collected from the patient charts. Survival, post-operative complications as well as the functional status (Karnofsky/Lansky scale) at the end of follow-up were measured.

**Results:**

In total, 87 patients were listed for HT, of whom 19 (22%) died while on the waiting list. Four patients were removed from the waiting list and 64 (74%) underwent transplantation. Median recipient age at HT was 12.0 (IQR 7.2–14.4) years old; 55% were female. One-, 5‑, and 10-year survival post-HT was 97%, 95%, and 88%, respectively. Common transplant-related complications were rejections (50%), Epstein-Barr virus infections (31%), cytomegalovirus infections (25%), post-transplant lymphoproliferative disease (13%), and cardiac allograft vasculopathy (13%). The median functional score (Karnofsky/Lansky scale) was 100 (IQR 90–100).

**Conclusion:**

Children who undergo HT have an excellent survival rate up to 10 years post-HT. Even though complications post-HT are common, the functional status of most patients is excellent. Waiting list mortality is high, demonstrating that donor availability for this vulnerable patient group remains a major limitation for further improvement of outcome.

## What’s new?

- Mortality in paediatric patients on the waiting list for heart transplantation (HT) is high (22%).
- Waiting list mortality significantly decreased after the introduction of paediatric ventricular assist devices in 2007 (41% before 2007 vs 17% after 2007, *p* = 0.03).
- The survival rate post-HT is excellent with a 1-, 5‑, and 10-year survival of 97%, 95%, and 88%, respectively.
- Common complications post-HT are rejections (50%), Epstein Barr virus infections (31%), cytomegalovirus infections (25%), post-transplant lymphoproliferative disease (13%), and cardiac allograft vasculopathy (13%).
- Even though comorbidities are common, the functional status of patients is good with a median Karnofsky/Lansky score of 100.

## Introduction

Heart transplantation (HT) is a widely accepted treatment option for selected adults and children with end-stage heart failure refractory to medication [1, 2]. In children, only 600–700 HTs are performed each year by approximately 120 centres worldwide [2]. Therefore, most centres perform only a limited number of procedures annually with 73% of centres performing 1–4 paediatric HTs a year [3]. In Europe, the majority of centres report < 5 HTs/year, whereas the majority of centres in North America report > 10 HTs a year [2, 3]. Beyond infancy, (dilated) cardiomyopathy is the most important indication for HT in Europe (55%), while in North America cardiomyopathies and congenital heart disease (CHD) are both seen in 40% of patients[2]. Waiting list mortality is high with percentages reported between 18% and 40% despite the use of mechanical circulatory support (MCS), including paediatric ventricular assist devices (VADs) [4–7]. However, in children VADs are frequently associated with bleeding and thromboembolic events [7–9].

In the Netherlands, a paediatric HT programme was initiated at the Erasmus MC—Sophia Children’s Hospital in 1998. Since then, it has been serving as the national centre for end-stage heart failure and HT in children. Here, we report the outcomes of children listed for HT since 1998.

## Methods

### Patients

All children listed for HT at our centre between 1998 and October 2020 were included. Follow-up lasted until death, retransplantation, or 1 January 2021 (end of follow-up), whichever came first. Listing criteria were applied according to the International Society for Heart and Lung Transplantation (ISHLT) guidelines [1]. Use of MCS was registered. Primary endpoints were waiting list outcome (mortality, transplantation, delisting) and survival post-HT. Moreover, transplant-related complications were examined, including: (acute) rejection episodes, cardiac allograft vasculopathy (CAV), infections, malignancies (solid-organ malignancies, post-transplant lymphoproliferative disease (PTLD) and skin malignancies), kidney failure, and other complications. The functional status of the patient was determined by the 100-point Karnofsky/Lansky scale at the end of follow-up [10–12]. The study was approved by the local medical ethics committee (MEC 2018–1348).

### Donors

Collected donor characteristics included donor age, body weight, blood type, and total ischaemia time.

### Heart transplantation procedure

All hearts transplanted were from ABO-compatible donors. The surgical procedure included a biatrial anastomosis; in patients with CHD a bicaval anastomosis technique could be used.

### Immunosuppression

The immunosuppression consisted of induction with anti-thymocyte globulin (ATG), followed by triple therapy: before 2000, patients were treated with (1) steroids and successive tapering in the 1st year, (2) cyclosporine, and (3) azathioprine. After 2000, tacrolimus replaced cyclosporine and mycophenolate mofetil replaced azathioprine. Rejection therapy consisted of pulse-dose methylprednisolone (10–15 mg/kg), occasionally followed by ATG in the case of an insufficient response to methylprednisolone.

### Graft surveillance

Surveillance endomyocardial biopsies were performed at: week 1–2, 3–4, 6, and 12, month 4–5, 6, 9, and 12 and whenever rejection was suspected. Rejection was graded according to the ISHLT classification: grades 2R and higher were considered relevant. Classifications before 2005 were revised according to the ISHLT guidelines [13]. CAV was graded according to the ISHLT guidelines and evaluated 1 and 2 years post-HT with coronary angiography and subsequently every 2 years by CT angiography or coronary angiography [14, 15].

### Definitions

Hypertension was defined as the use of antihypertensive drugs, excluding the period immediately post-transplant. Cytomegalovirus (CMV) and Epstein-Barr virus (EBV) infections were defined as viral loads of > 1000 copies/ml irrespective of clinical symptoms or, in the presence of symptoms, with viral loads ≤ 1000 copies/ml.

### Statistical analysis

Categorical variables are reported as numbers and percentages. Continuous variables are reported as means with standard deviation (SD) when normally distributed, and medians with 25th–75th percentile (interquartile range (IQR)) otherwise. Continuous variables were compared by Student’s *t*-tests (normal distribution) or Mann-Whitney U tests. Categorical analysis was conducted by χ^2^ and Fisher’s exact test. Time on waiting list and survival after HT were estimated using Kaplan-Meier curves and the log-rank test. All analyses were performed using IBM SPSS Statistics for Windows, version 25 (IMB Corp., Armonk, NY, USA).

## Results

### Patients

Eighty-seven patients were listed for HT at the age of 10.6 (IQR 3.0–13.9) years; 48 (55%) were female. Aetiology was dilated cardiomyopathy (64%), restrictive cardiomyopathy (13%), CHD (6%), and other (17%). Blood groups O (45%) and A (43%) were most common. Time from diagnosis to HT listing was 16 (IQR 3–57) months and 31% had a VAD pre-HT. Baseline characteristics are summarised in Tab. 1.

**Table 1:** Baseline characteristics of paediatric patients listed for heart transplantation during the study period (*n* = 87). Categorical variables are presented as absolute numbers with (percentages), continuous variables as medians and 25th–75th percentile (interquartile range)

Parameters	
Age (years) at listing	10.6 (3.0–13.9)
– < 1 year old	10 (12)
– 1–10 years old	29 (33)
– ≥ 10 years old	48 (55)
Female	48 (55)
*Diagnosis*	
– Congenital heart disease	5 (6)
– Dilated cardiomyopathy	56 (64)
– Hypertrophic cardiomyopathy	3 (3)
– Restrictive cardiomyopathy	11 (13)
– Non-compaction cardiomyopathy	8 (9)
– Chemotherapy-induced cardiomyopathy	4 (5)
Time from diagnosis of HF to listing for HT	1.4 (0.3–4.8)
– Listed within 1 year after diagnosis	37 (43)
*Eurotransplant status at listing*	
– Hospitalised	63 (72)
– At home	24 (28)
MCS on waiting list	27 (31)
– Berlin Heart	15 (56)
– Levitronix	11 (41)
– ECMO	1 (4)
*Blood group*	
– A	37 (43)
– B	8 (9)
– AB	3 (3)
– O	39 (45)

*ECMO* extracorporeal membrane oxygenation, *HF* heart failure, *HT* heart transplantation, *MCS* mechanical circulatory support

### Waiting list outcome

**Fig. 1:**
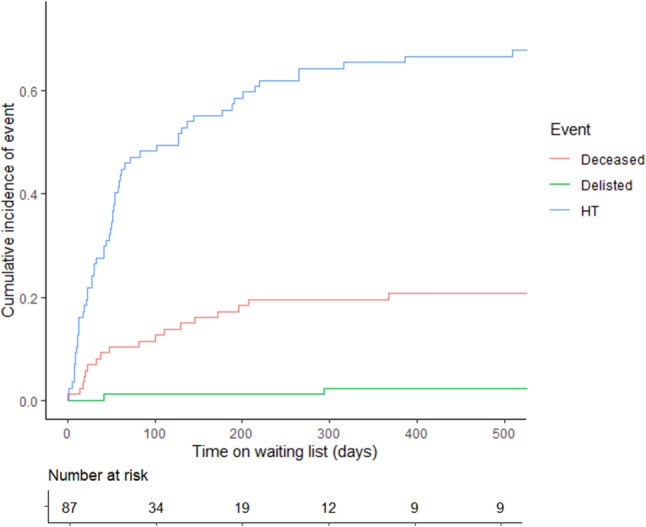
Incidence of outcomes while on the waiting list (delisted, died or heart transplantation). *HT* heart transplantation

**Fig. 2:**
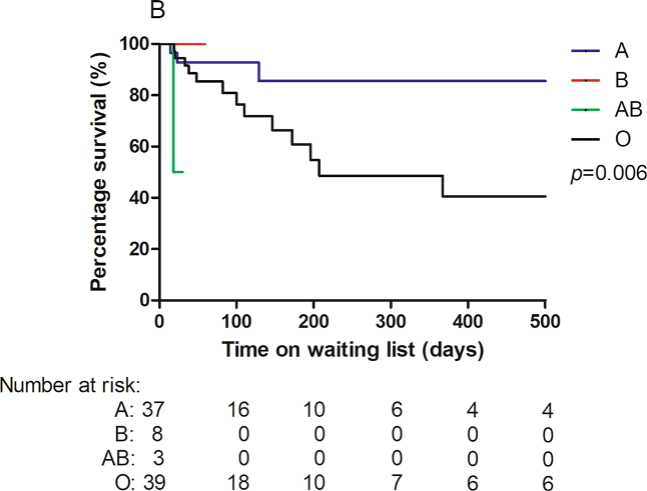
Waiting list mortality for paediatric patients listed for heart transplantation stratified by blood group

Time on the waiting list was 54 (IQR 21–188) days. Nineteen (22%) patients died, 4 (4%) were delisted, and 64 (74%) underwent HT. Delisted patients improved (*n* = 2) or had a worsening condition prohibiting HT and subsequently died (*n* = 2). Waiting list mortality was associated with younger age (2.1 (IQR 0.9–11.6) years) and blood group O (80%). Reasons for death were VAD-related complications (47%) necessitating withdrawal of support and end-stage heart failure (53%). Patients who were listed within 1 year after diagnosis were more likely to be hospitalised (84% vs 64%, *p* = 0.04), on MCS (49% vs 18%, *p* = 0.002), and died more often while on the waiting list (35% vs 12%, *p* = 0.01). Seventeen patients were listed before the introduction of VADs in 2007, and 70 after 2007. Waiting list mortality decreased significantly after 2007 (41% vs 17%, respectively, *p* = 0.03). Patients with blood groups A and B had better outcomes than those with blood groups AB and O (*p* = 0.006) (Figs. 1 and 2).

### Heart transplantation

Sixty-four children underwent HT at 12.0 (IQR 7.2–14.4) years and the majority (58%) were hospitalised before HT. Ischaemia time was 222 ± 46 min. Donors were 15 (IQR 9–25) years old. Donor and recipient characteristics are summarised in Tab. 2.

**Table 2:** Baseline characteristics of paediatric patients who underwent a heart transplantation (*n* = 64). Categorical variables are presented as absolute numbers with (percentages), continuous variables as means ± standard deviation when normally distributed or medians with 25th–75th percentiles (interquartile range) when not normally distributed

Parameters	
*Donor*	
Age (years)	15 (9–25)
Female	34 (53)
BMI	20.5 (17.4–24.0)
*Country of origin*	
– Netherlands	14 (22)
– Germany	31 (48)
– Belgium	10 (16)
– Other	9 (14)
*Recipient*	
Age (years) at HT	12.0 (7.2–14.4)
– < 1 year old	2 (3)
– 1–10 years old	17 (27)
– ≥ 10 years old	45 (70)
Female	35 (55)
Time on waiting list (days)	52 (20–169)
Ischaemia time (min)	222 ± 46
*Eurotransplant status at HT*	
– Hospitalised	37 (58)
– At home	27 (42)
*Blood group*	
– A	32 (50)
– B	8 (13)
– AB	2 (3)
– O	22 (34)
Induction therapy with ATG	64 (100)

*ATG* anti-thymocyte globulin, *BMI* body mass index, *HT* heart transplantation

**Fig. 3:**
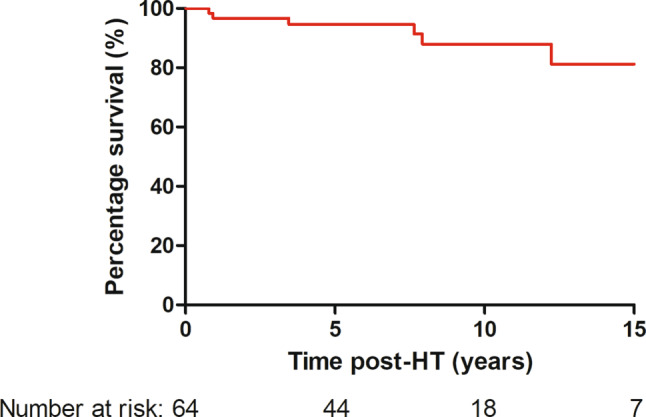
Overall survival following heart transplantation (*HT*)

Follow-up duration after HT was 7.4 (IQR 3.1–10.5) years. Use of immunosuppressants at different time points is shown in Tab. 3. The 1‑, 5‑, and 10-year survival was 97%, 95%, and 88%, respectively (Fig. 3). One patient underwent retransplantation 15.6 years after the first HT due to right-sided heart failure secondary to long-standing tricuspid valve regurgitation, following unsuccessful tricuspid valve repair.

**Table 3:** Immunosuppressant use at discharge, 1 year after heart transplantation (*HT*) and at last follow-up. All data are presented as absolute numbers with (percentages)

Immunosuppressant	At discharge (*n* = 64)	One year post-HT (*n* = 58)	At last follow-up (*n* = 64)
Tacrolimus	58 (91)	56 (97)	64 (100)
Cyclosporine	6 (9)	2 (3)	0 (0)
Mycophenolate mofetil	59 (92)	29 (50)	26 (41)
Prednisolone	64 (100)	30 (52)	19 (30)
Everolimus	0 (0)	4 (7)	6 (9)
Azathioprine	1 (2)	1 (2)	0 (0)
Sirolimus	0 (0)	0 (0)	2 (3)
Monotherapy	0 (0)	13 (22)	21 (33)

### Rejections

Patients underwent a median of 10 (IQR 9–14) biopsies. Rejections were found in 32 (50%) patients with a median of 1 (IQR 0–2) rejection per patient. Time to first rejection was 30 (IQR 11–111) days. Between discharge and the end of the 1st year post-HT, 31% of patients developed at least one rejection episode. Three patients had severe rejections with compromised haemodynamics; 2 patients had a histologically proven severe (grade 3R) rejection. The third patient was too unstable to undergo a biopsy. One could be treated with intravenous methylprednisolone only; the second patient needed inotropic support as well. The third patient developed biventricular failure requiring extracorporeal membrane oxygenation. Two out of 3 patients had been non-compliant with their medication. Overall, non-compliance was demonstrated in 8 (13%) patients during follow-up.

### Infections

EBV and CMV infections were found in 20 (31%) and 16 (25%) patients, respectively. Time between HT and EBV infection was 5 (IQR 4–10) months, for CMV infection 4 (IQR 1–6) months. Other infections included herpes zoster (14%), *Candida* (5%), *Aspergillus* (3%), *Pneumocystis jirovici* (3%), and severe acute respiratory syndrome coronavirus 2 (SARS-CoV-2) infections (3%).

### Renal function, diabetes and hypertension

Acute kidney injury occurred in 29 (45%) patients post-HT, of whom 4 (6%) needed temporary renal replacement therapy. In 2 patients (3%) kidney failure (estimated glomerular filtration rate < 15 ml/min per 1.73 m^2^ and/or renal replacement therapy) occurred during end-stage heart failure of the donor heart. No patient developed kidney failure due to chronic calcineurin inhibitor use. Diabetes mellitus was seen in 7 (11%) patients, of whom 5 (8%) were insulin-dependent. Hypertension was present in 28 (44%) patients at the end of follow-up.

### Malignancies

Eight (13%) patients developed PTLD at a median of 5 (IQR 4–7) months post-HT. All cases were related to EBV infections. Six patients were treated with rituximab, 1 by lowering immunosuppression. One patient developed a full-blown lymphoma and required extensive chemo-radiotherapy. This patient has remained in remission for more than 5 years after the end of treatment. In 1 patient, a melanoma was successfully treated by local resection.

### Cardiac allograft vasculopathy

CAV developed in 8 (13%) patients. CAV grade 1, 2, and 3 were seen in 2 (3%), 2 (3%), and 4 (6%) patients, respectively. Patients with CAV grade 2 or 3 were mostly treated with medication adjustments and stents. In 1 patient, CAV 3 was diagnosed at autopsy after sudden death within the 1st year post-HT. Three out of 8 patients (38%) with CAV had been non-compliant during follow-up.

### Other

Neurological complications were seen in 9 patients (14%), including posterior reversible encephalopathy syndrome (*n* = 3), epileptic insult (*n* = 3), peripheral neuropathy (*n* = 1), cerebrovascular accident (*n* = 1), and transient ischaemic attack (*n* = 1).

### Performance status

On the Karnovsky/Lansky scale, at the last follow-up visit, patients scored 100 (IQR 90–100) points, indicating that the majority of patients were able to perform normal daily activities.

## Discussion

In this study, we report 23 years’ experience of a national programme for paediatric HT in the Netherlands. In line with ISHLT registry reports, it is a small- to medium-sized programme [2, 3]. Waiting list mortality (22%) was high, but outcomes were excellent in those who reached transplantation, with a 1‑, 5‑, and 10-year survival of 97%, 95%, and 88%, respectively, and overall good functional outcomes.

Waiting list mortality in our study was high (22%) but is in line with previous studies, which have reported a waiting list mortality of 18–40% [4–6]. Blood type significantly influenced waiting list outcome, which is in line with Eurotransplant experience [6], even though this was not seen in a North American study [16]. In a previous study, we reported a low rate of listing and transplantation in children in the 1st year after presentation as compared to several other registries [17]. There was no increase in early mortality, nor in transplantation rate in subsequent years [17]. Our strategy to reserve listing early after presentation for the sickest children is underscored by the characteristics of those who were listed within 1 year of presentation, with high rates of hospitalisation (84%) and VAD support (49%). Mortality in patients listed within 1 year was high (35%), despite the fact that as of 2011 all children (< 16 years) within Eurotransplant are listed with a high urgency status and those who are hospitalised are prioritised and have the highest international urgency status (IHU) [6]. Before 2011, an IHU had to be specifically requested and was granted for all hospitalised children [6]. In our study, the introduction of VAD support in 2007 had a major impact on waiting list mortality. Since then, waiting list mortality has decreased from 41% to 17% and was mostly related to VAD support complications [8].

Survival in our cohort compares favourably to that of other single-centre studies with reported 1‑, 5‑, and 10-year survival of 83–92%, 74–82%, and 63–80%, respectively [4, 5, 18–22]. Similarly, the ISHLT registry reported 1‑year survival of 92% and a 5-year survival, in those who survived the 1st year post-HT, of 91% in the most recent era [2]. Of note is that HT for cardiomyopathy has better outcomes than CHD [2]. In our cohort, cardiomyopathy was the main indication for transplantation but, even when taking this into account, the outcome of our patients is at least comparable with international reported data [2, 20, 21].

Rejection is common post-HT [3] and 50% of our patients had at least one rejection, of which 31% occurred between discharge and the 1st year post-HT. This is in line with other single-centre studies [4, 5, 21], while the ISHLT demonstrated a rejection incidence between discharge and 1 year post-HT of 13–24% [3]. A possible explanation for this could be that younger patients are at a lower risk of developing rejections, and in our cohort the median age was significantly higher than that of those included in the ISHLT registry [3]. PTLD is also a common complication post-HT with the ISHLT reporting an incidence in children of 11% after a follow-up of 10 years. Skin and solid-organ malignancies are rarely seen in children [2, 3]. This is in accordance with our study [3]. Finally, CAV is an important complication with a high morbidity and mortality rate with an incidence in the literature between 20% and 40% at 10 years and 50% at 15 years post-HT [3, 5, 19–21]. Even though our results seem favourable with an incidence of 13% at 7.4 years, definite conclusions on the incidence in our cohort cannot be drawn yet.

A major concern in paediatric HT recipients is non-compliance to medication, with adolescent recipients at the highest risk [20, 23]. This has been suggested as one of the reasons why patients between 10 and 18 years old have an impaired survival compared to younger age groups [2]. Our study also suggests that non-compliance may significantly increase the risk of severe rejections and the development of CAV. It is essential to support patients during adolescence and to emphasise the importance of compliance to medication.

Our study has several limitations. First, we report a retrospective analysis of a single-centre, small- to medium-sized paediatric programme. However, it demonstrates that by concentrating experience in one centre nationwide, closely cooperating with referring hospitals and by combining the programme with adult HT experience, this treatment option can be offered with good outcomes. Furthermore, the number of patients surviving more than 10 years is still limited. Thus, our results are mainly a reflection of short- to medium-term outcome.

In conclusion, we demonstrate that HT in children can be performed with good survival and functional outcome, by concentration of the experience in one nationwide programme of relatively limited size. As in adults, donor availability for this vulnerable group remains a major limitation for further improvement of outcome.
